# Ediacara biota flourished in oligotrophic and bacterially dominated marine environments across Baltica

**DOI:** 10.1038/s41467-018-04195-8

**Published:** 2018-05-04

**Authors:** Kelden Pehr, Gordon D. Love, Anton Kuznetsov, Victor Podkovyrov, Christopher K. Junium, Leonid Shumlyanskyy, Tetyana Sokur, Andrey Bekker

**Affiliations:** 1 Department of Earth Sciences, University of California, Riverside, 900 University Avenue, Riverside, CA 92521 USA; 2 Institute of Precambrian Geology and Geochronology, RAS, nab. Makarova 2, St. Petersburg, 199034 Russia; 3 Department of Earth Sciences, Syracuse University, 322 Heroy Geology Lab, Syracuse, NY 13244 USA; 4 M.P. Semenko Institute of Geochemistry, Mineralogy and Ore Formation, National Academy of Sciences of Ukraine, 34 Palladina Av, Kiev, 03142 Ukraine; 5 Institute of Geological Sciences, National Academy of Sciences of Ukraine, Olesya Honchara Str., 55-b, Kiev, 01054 Ukraine

## Abstract

Middle-to-late Ediacaran (575–541 Ma) marine sedimentary rocks record the first appearance of macroscopic, multicellular body fossils, yet little is known about the environments and food sources that sustained this enigmatic fauna. Here, we perform a lipid biomarker and stable isotope (δ^15^N_total_ and δ^13^C_TOC_) investigation of exceptionally immature late Ediacaran strata (<560 Ma) from multiple locations across Baltica. Our results show that the biomarker assemblages encompass an exceptionally wide range of hopane/sterane ratios (1.6–119), which is a broad measure of bacterial/eukaryotic source organism inputs. These include some unusually high hopane/sterane ratios (22–119), particularly during the peak in diversity and abundance of the Ediacara biota. A high contribution of bacteria to the overall low productivity may have bolstered a microbial loop, locally sustaining dissolved organic matter as an important organic nutrient. These oligotrophic, shallow-marine conditions extended over hundreds of kilometers across Baltica and persisted for more than 10 million years.

## Abstract (web summary)

The environments and food sources that sustained Ediacara biota 575-541 million years ago remain unclear. Here, the authors perform lipid biomarker and isotopic analyses on biota fossil-containing Ediacaran strata from Baltica and propose the presence of a microbial loop bolstered by bacteria.

## Introduction

The Ediacaran Period (~635–541 Ma) was part of an era of extreme tectonic, geochemical, and evolutionary changes, which fundamentally reorganized marine ecosystems. Oxygen has often been proposed as an enabler, if not the driver, of the rise and evolution of multicellular biota and metazoans. Trace-metal and isotope redox proxies place an atmospheric and ocean surface oxygenation event at ~850 Ma^1,2^, and numerous settings of oxygenated shallow waters have been identified throughout the Ediacaran^3–5^. The Ediacaran is known for its wide variety of fossils, notably body and trace fossils, left by soft-bodied multicellular fauna unique to the middle and late Ediacaran (ca. 575–541 Ma^6,7^). While there are also anoxic, and even euxinic, productive marine settings identified at this time^5,8^, redox proxies in the majority of units containing the diverse and large Ediacara biota indicate predominantly oxic conditions^9^. The emergence of metazoan fauna at that time has thus often been ascribed to increasing oxygenation of the oceans. This interpretation has been challenged on the basis that anatomically simple metazoans, particularly sponges, could survive under very low dissolved oxygen levels, which may have been sustained in shallow waters prior to the Ediacaran^10^. However, detailed geochemical studies, in some cases across a range of ecosystems, do show a correlation between oxygen stability and fossil diversity and size^4,9^. It seems most likely that while metazoans might have initially evolved in low-oxygen environments, it was not until oxygen level increased enough to support persistent oxygenation of shallow waters that the macroscopic Ediacara biota began to flourish^11,12^.

However, oxygen was likely not the only control on metazoan early evolution and diversification. Nutrient availability, usually of nitrogen or phosphorus, limits the productivity and composition of microbial communities and would have imposed an important selective pressure on the Ediacara biota. It has been proposed that marine anoxia in the Proterozoic Era prior to the late Ediacaran could have favored nitrogen limitation in the oceans by supporting denitrification and anammox reactions and thus limited metazoan evolution and expansion^13^. However, as oxygenated surface waters expanded globally in the late Neoproterozoic^5,11,14^, nitrogen limitation would have become less persistent, and productivity on shelves became increasingly controlled by advection of nutrients, resulting in productivity patterns more similar to those in Phanerozoic oceans^15^. Ferruginous deep waters in the Archean and early Proterozoic likely also limited bioavailable phosphorous^16^. While seawater phosphate concentration probably increased substantially due to a fundamental shift in the phosphorus cycle during the Cryogenian period associated with the glaciations and their aftermaths^16,17^, the temporal trajectory of phosphate marine availability through the late Ediacaran is not readily deciphered and was likely variable from location to location. The role that essential nutrients and food sources, including carbon, nitrogen, and phosphorus, played in the early evolution and diversification of metazoans during the Ediacaran remains open for debate and deserves further study.

On the basis of their morphology, some of the earlier Ediacara biota are thought to have used osmotrophy, which is the uptake of dissolved organic compounds by diffusion, while other types of the Ediacara biota are thought to show evidence of suspension feeding and active (motile) heterotrophy^18–20^. The Ediacara biota is traditionally divided into three groups on the basis of their age, diversity, and unique biological and ecological capabilities^20^. Exact age constraints are however scarce, and the fossil records of these groups seem to overlap in some locations. The oldest, Avalon assemblage is usually associated with deep-water settings and high body surface area to volume ratios, characteristic of osmotrophy^21^. The second group, the White Sea assemblage, is notable for morphologies that suggest increased mobility, bilaterian forms, and evidence of burrowing and skeletonization^22–25^. The third group, the Nama assemblage, maintained many of the evolutionary innovations observed in the White Sea assemblage, but is characterized by a sharp decrease in diversity. Lack of taphonomically suitable environments and changes in environmental conditions, as well as increased competition from evolving predators have been proposed as a cause for the disappearance of many forms of the Ediacara biota present in the White Sea assemblage from the Nama assemblage^26,27^. Near the end of the Ediacaran, the first calcified metazoans appeared, which has been suggested to result in a positive feedback between more oxygenated oceans and the rate of organic matter export due to their higher density^28^.

In addition to the body fossil record, lipid biomarker assemblages consisting of molecular fossils derived from cell membrane lipids and other recalcitrant organic molecules can be used to investigate source organism inputs and local paleoenvironmental redox conditions^29^. Organic-matter-rich deposits are typically targeted for biomarker analysis due to their high potential of preserving sufficient extractable material for detection, however, organic-matter-lean deposits can also preserve abundant biomarkers if they have undergone only a mild thermal alteration. Diverse and abundant lipid biomarker assemblages have been previously reported from a variety of Ediacaran, eutrophic marine environments from rocks and/or oils of appropriate thermal maturity from South Oman^30,31^, India^32^, eastern Siberia^33,34^, Australia^35^, and Russia^36^.

**Fig. 1:**
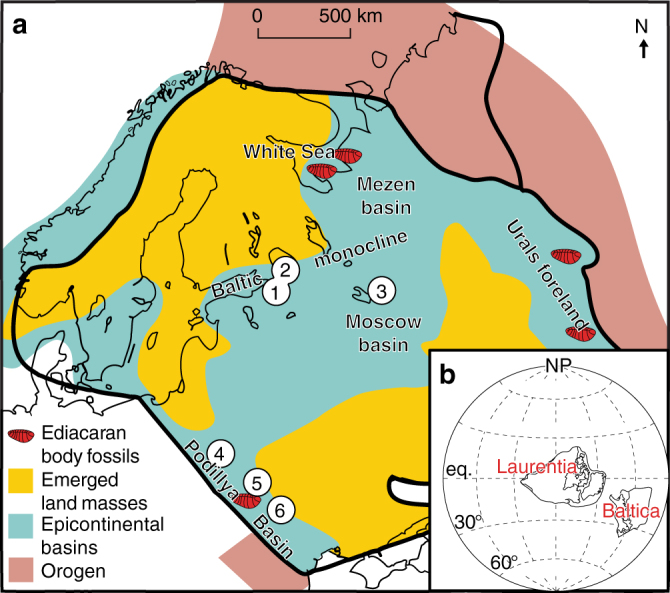
Paleogeography and known Ediacara biota fossil occurrences across Baltica. **a** Paleogeographic reconstruction of Baltica during the late Ediacaran (modified from Sliaupa et al.^83^) with studied drill cores and outcrops shown (1—Utkina Zavod; 2—Lugovoe; 3—Gavrilov-Yam-1 in Russia; 4—4504, 4529, and 4592 cores from the Volyn Basin, Ukraine; 5—3628 core from the Podillya Basin, Ukraine; and 6—outcrops 16PL from the Podillya Basin, Moldova). **b** Global reconstruction of Laurentia and Baltica at ~550 Ma, modified from Fedorova et al.^84^

Here, we present the lipid biomarker and nitrogen and carbon isotopic data obtained from exceptionally immature Ediacaran strata from seven drill cores and three outcrops spanning Baltica. Two of the drill cores, Utkina Zavod and Lugovoe, were located near St. Petersburg in the northeastern part of the Baltic monocline. Gavrilov-Yam-1 was drilled in the Moscow Basin, the 4504, 4529, and 4592 drill cores were recovered from the Volyn region of Ukraine, while the 3628 drill core and 16PL outcrops of Podillya Basin were sampled in southwestern Ukraine and Moldova, respectively (see Fig. 1 and Supplementary Table 1). Ediacara biota fossils have been described extensively across Baltica, as shown in Fig. 1, including abundant occurrences of White Sea-type fossil assemblages from the Redkino Horizon in Podillya outcrops^37^, close to where our own Podillya samples were collected. We studied fine-grained sedimentary rocks (mudstones and siltstones) of the Redkino and Kotlin Horizons from these sites; in addition, the Lontova Horizon (*Platysolenites antiquissimus* Zone) of the early Cambrian age was sampled in the Gavrilov-Yam-1 drill core.

## Results and discussion

### Exceptionally low thermal maturity of the strata

**Fig. 2:**
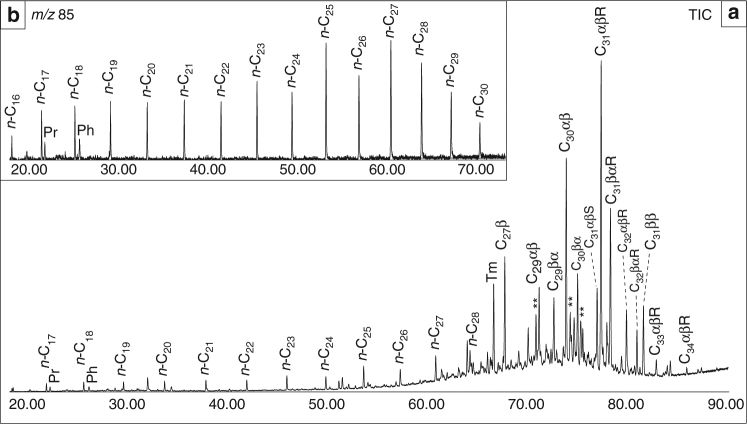
Distribution of extractable aliphatic hydrocarbons for a representative sample. **a** Total ion chromatogram (TIC) for extractable aliphatic hydrocarbons for Lugovoe #13–73 m from the Redkino Horizon. The *n*-alkane series, pristane (Pr), phytane (Ph), and C_27_–C_34_ hopanes (denoted by their total carbon number and stereochemistry at C-17, C-21, and C-22, e.g., C31αβR) are labeled; ** denotes C_29_ and C_30_ hopenes. Note the hopane abundance dominance over other alkane compound classes. **b** Partial 85-Da ion chromatogram shows dominance of the *n*-alkane series, exhibiting a discernible odd-over-even carbon-number preference in the C_22_–C_27_ range, over methylalkanes

Thermal maturity is the single most important factor influencing the preservation of ancient sedimentary biomarkers as organic molecules are susceptible to structural and stereochemical alteration during progressive burial and with increasing thermal stress^29^. Precambrian rocks of appropriate thermal maturity (oil window maturity or lower) are prerequisites for preserving robust and primary biomarker lipid assemblages. Multiple hopane and sterane biomarker stereoisomer ratios, along with independent evidence from low *T*_max_ values (mostly within a 417–433 °C range, with a mean of 426 °C) from Rock-Eval pyrolysis, indicate that the rocks in this study represent by far the most thermally immature Ediacaran rocks analyzed to date using the state-of-the-art organic geochemical methods (Table 1). Although our rocks have undergone sedimentary diagenesis, they did not pass significantly into the oil window apart from the slightly more mature 16PL outcrop samples from Podillya Basin in Moldova, which are still suitable for analysis (early-to-middle oil window maturity and no obvious sign of organic contaminants). The majority of the set then had not likely been exposed to burial temperatures exceeding 50 °C, thus our samples are highly immature. Other lines of molecular evidence, which support low thermal maturity and syngenicity, include (i) a dominance of polycyclic biomarker alkanes over *n*-alkanes in rock extracts (Fig. 2), (ii) survival of detectable amounts of 17β,21β(H)-hopanes resolvable from the more abundant hopanes possessing stable 17β,21α(H)- and 17α,21β(H)-stereochemical configurations (Table 1, Supplementary Figs. 1 and 2), (iii) a discernible odd-over-even preference among the *n*-alkanes in the C_22_ to C_27_ range (Fig. 2), as *n*-alkanes show a carbon-number preference only prior to catagenesis^38^, and (iv) generation of thermally immature hopane and sterane biomarkers from the (insoluble) kerogen phase using catalytic hydropyrolysis (Supplementary Fig. 2). The low thermal maturity of sedimentary organic matter in our samples is consistent with previously published indicators of sedimentary alteration, including conodont and acritarch alteration indices, and previous Rock-Eval pyrolysis and biomarker studies of the Ediacaran and Paleozoic sedimentary rocks in Baltica^36,39,40^, as well as clay mineralogy^41^. Critically, in settings where thermal maturity is this low, the mechanisms for significant alteration of primary δ^15^N and δ^13^C signals are largely absent^42^.

**Table 1:** Select lipid biomarker ratios for thermal maturity, source biota, and depositional environmental assessments

^a^Hop/Ster is the ratio of major (C_27_–C_35_ hopane isomers)/(C_27_–C_30_ diasteranes and regular steranes)

^b^C_31_ 2-methylhopane index (2-MeH index) calculated as [(C_31_ 2α-methylhopane+C_31_ 2β-methylhopane)/(C_31_ 2α-methylhopane+C_31_ 2β-methylhopane+C_30_ αβ hopane)*100]

^c^C_31_ 3-methylhopane index (3-MeH index) calculated as [(C_31_ 3β-methylhopane)/(C_31_ 3β-methylhopane+C_30_ αβ hopane)*100]

^d^Relative percent of C_*n*_ steranes to total C_27_–C_30_ steranes

^e^24-isopropylcholestane abbreviated as C_30_ ipc

^f^24-*n*-propylcholestane abbreviated as C_30_ npc

^g^Not detected (n.d.) indicates that the peaks were below MRM–GC–MS detection limits due to negligible abundance

### Lipid biomarker assemblage patterns

An abundance ratio of the major (C_27_–C_35_) hopanes to major (C_27_–C_29_) steranes is often used to assess the balance of bacterial versus eukaryotic source organism inputs to the ancient aquatic ecosystem. Hopanes are molecular fossils derived from hopanoids, which are cell membrane lipids synthesized by a wide variety of bacterial groups. Similarly, steranes are derived from sterol precursors, which are produced almost exclusively by eukaryotes^43^. Hopane/sterane (H/St) ratios for our samples cover a strikingly large range of values from 1.6 to 119.2 (Table 1). For context, H/St ratios from organic-rich Neoproterozoic rocks and oils typically fall in a narrow range from 0.5 to 2.0^30–33^. While there is a clear difference in the values from the younger Kotlin Horizon (average H/St of 8.9) and the older Redkino Horizon (average H/St of 42.9), the values for most samples are unusually high and suggest anomalously elevated contributions of bacteria.

By the late Neoproterozoic, eukaryotic algae were an ecologically significant component and major producers in many marine ecosystems^15,34,44^. The discrepancy between the globally important contribution of eukaryotic algae in the late Neoproterozoic and yet the low levels of sterane biomarkers in these samples indicates that there must be some local determinant on eukaryotic abundance. The most parsimonious explanation for the extremely elevated hopane/sterane ratios alongside low total organic carbon (TOC) contents and low hydrogen indices (HI) found for our samples (Table 1) is that these strata were deposited in oligotrophic (i.e., strongly nutrient-limited) settings, in which bacteria outcompeted algae. While modern analogs for ancient epicratonic seas developed during high sea-level stand are hard to find, parallel observations of higher hopane/sterane ratios (by up to an order of magnitude) have been found previously for organic-lean versus organic-rich sedimentary rocks deposited in Ordovician–Silurian epicontinental seaways^45^.

Typically, low TOC content can be caused by limited deposition of organic matter in low-productivity settings, low preservational potential of organic matter in the water column or sediments, or dilution with a high siliciclastic flux. In modern oceans, productivity is most commonly limited by low levels of the essential nutrients: nitrogen, phosphorus, silicon, and iron^46^. Nitrogen, phosphorus, and iron are limited in open-ocean regions where upwelling, dust input, and coastal runoff do not supply sufficient amounts of nutrients. In modern oligotrophic settings, the ratio of bacterial to eukaryotic biomass is higher than in eutrophic or mesotrophic settings. Interestingly, the Podillya 16PL outcrop samples from the Redkino horizon of Moldova are phosphorite-containing mudstones and yield significantly lower hopane/sterane ratios (8.1:11.5) than the other Redkino samples in Table 1 (though still significantly higher than those found in South Oman Salt Basin or in organic-rich Phanerozoic sediments, which typically fall within a narrow, 0.5–2.0 range), possibly suggesting that increased phosphate availability could have favorably influenced the eukaryotic-to-bacterial ratio found locally (given that the nitrogen isotopic signatures are largely invariant, see the next section). Phosphorus (P) contents, as well as P/Fe_total_ and P/Al ratios, are otherwise generally low for the late Ediacaran sediments of Podillya, Ukraine, and Estonia^47^; with the exception of this stratigraphic level marked with phosphorite nodules. Similarly, low (~0.01 to ~0.1 wt%) levels of P in Kotlin and Redkino siliciclastic rocks were reported from a drill core from the northeastern margin of the East European Platform^11^.

The broad and shallow topography in the epicontinental seas across Baltica could have sustained phosphorus or other nutrient limitations in marginal settings, due to authigenic precipitation of phosphate with iron minerals in the oxic surface waters and sequestration of a range of elements by shelfal sediments^48^. If phosphate was a limiting nutrient, increasing bioavailable phosphorus would have enhanced local primary production and provided more favorable growth conditions for larger-sized unicellular phytoplankton^49–51^. With respect to the modern ocean system, the marine picocyanobacteria, *Prochlorococcus* and *Synechococcus*, are recognized to dominate phytoplankton cell counts and biomass in the oligotrophic tropical and subtropical ocean settings, including phosphate-limited oligotrophic regions of ocean-surface waters^52^. *Prochlorococcus* and the heterotrophic *SAR11* (Pelagibacter) flourish due to a variety of adaptations, including low-energy costs by virtue of small genomes and low replication rates, a higher surface-area-to-volume ratio through smaller cell sizes, and additional cell uptake functions to maximize nutrient utilization^53^. Many bacteria are also able to substitute low-abundance nutrients, e.g., by utilizing sulfolipids instead of phosphorus-bearing lipids in P-deficient settings^54^, or by use of alternative substrates, e.g., sourcing nitrogen from atmospheric N_2_ via nitrogen fixation^55^, to alleviate nutrient stress. A recent study regarding survival of marine bacterioplankton in oligotrophic environments^56^, where available phosphate is limited, suggests an important role for polyphosphate metabolism in marine oligotrophs. The select eukaryotes that compete in oligotrophic settings are typically small picoeukaryotes, which may supplement their nutritional requirements through mixotrophy^57^. Picoeukaryote-to-cyanobacteria biomass ratio tends to increase under enhanced nutrient supply^49^. The possible influence of phosphorus, and other biolimiting nutrients, in moderating primary productivity and marine community structure in Ediacaran epeiric seaways requires further investigation.

Without exception, the abundance of C_29_ steranes is greater than the corresponding C_27_ or C_28_ steranes for all our locations (Table 1). A predominance of C_29_ over C_27_ and C_28_ steranes likely indicates a green-algae dominance within the eukaryotic phytoplankton community^44,58^. This feature has been observed in most previous Ediacaran biomarker studies^15,30–36,44^. Notably, the C_30_ sterane distribution in several samples from each drill core in the Kotlin Horizon contains low, but detectable, amounts of the demosponge sterane biomarker known as 24-isopropylcholestane (24-ipc)^59,60^. In total, 24-ipc steranes have been reported in rock and oil samples dating as far back as the Cryogenian (>635 Ma ago) in the South Oman Salt Basin and represent the oldest lipid biomarker evidence for metazoans^59,60^. The 24-ipc biomarkers in our samples were either around one order of magnitude lower in abundance (relative to the total C_27_ to C_30_ sterane ratios, these were only 0.06–0.61%; mean = 0.22%) compared with Ediacaran rocks and oils from South Oman (1.7% on average^59^) or were below detection limits due to negligible abundance for the majority of samples.

### Nitrogen and organic carbon isotope ratios

Nitrogen isotopes can help discern the relative balance in the nitrogen cycle, and the degree to which either nitrogen fixation or incomplete denitrification were significant pathways to influence the nutrient balance available for marine communities. When diazotrophic bacteria fix molecular nitrogen due to a lack of fixed nitrogen in the water column, this can yield sedimentary bulk nitrogen isotope values near 0‰^61^. This is in contrast to the positive nitrogen isotope signatures (in the range of +2 to +10‰) with a mode of +4 to +6‰ found for the Neoproterozoic marine sediments deposited under what are thought to be nitrate-replete conditions where nitrate has only undergone partial denitrification^62^. Nitrogen isotope values for all of our samples bar one outlier (Table 1) cover a limited positive range from +3.5 to +6.5‰, which overlaps with the mode for late Neoproterozoic organic-matter-rich sedimentary rocks^62^. Constrained by redox proxies suggesting that oxic water- column conditions prevailed, our data imply that nitrate dominated the dissolved inorganic nitrogen pool. The range of δ^15^N values also suggests that N_2_-fixation was not the primary mode of nitrogen acquisition for primary producers. Rather, the nitrogen cycle was likely dominated by water-column recycling, and organic N was subject to quantitative oxidation to nitrate as it is in modern, proximal marine settings. Benthic denitrification and organic N burial would have been the primary sinks for dissolved inorganic nitrogen, and the range of δ^15^N values suggests only a limited role for incomplete water-column denitrification, which typically results in significant ^15^N-enrichment^63^. The limited variability in δ^15^N values therefore likely reflects δ^15^N of nitrate advected onto the platform, with minor influence from limited water column nitrate reduction and N_2_-fixation. Without strong δ^15^N evidence for nitrogen fixation, we hypothesize that nitrogen was not the primary biolimiting nutrient in the epicontinental basins of Baltica. Isolation from riverine and eolian sources of phosphorus, and oxic conditions in the broad, shallow-marine epicontinental basins may have enhanced the removal of authigenic phosphorite and trace-metal-bearing phases, resulting in nutrient limitation that constrained eukaryotic cell growth and production.

Total organic carbon (C_TOC_) isotope values range from –23.0 to –33.9‰, with the largest differences observed between the different drill-core locations. The relative ^13^C-enrichment to isotope ratios higher than ca. –28‰ contrasts with data from contemporaneous strata deposited in the eutrophic, open-marine settings of Oman^30,31^. However, the range we report is generally consistent with the δ^13^C_TOC_ range for other locations from Baltica deposited over the same time period^11^. The difference between Baltica and Oman might highlight the fact that δ^13^C_TOC_ values do not exclusively reflect a uniform secular change in the carbon cycle during this interval of time^31,64^. Rather, the δ^13^C_TOC_ range from Baltica may, in part, reflect the bacterially dominated microbial ecology suggested by the unique lipid biomarker ratios. Small-cell size, high surface-area-to-volume ratios, and slow growth rates under oligotrophic conditions can increase the magnitude of the fractionation during autotrophy (*ε*_p_)^65,66^, resulting in low δ^13^C values.

The mechanism for the more ^13^C-enriched isotopic signatures within the range reported^11^ for the late Ediacaran Baltica succession is less clear. The potential contribution of detrital, metamorphically altered organic matter to Precambrian low-TOC sedimentary successions is a possible mechanism for disparities between sites in δ^13^C_TOC_^64^. However, the TOC content of our samples, while low, is generally greater than 0.10 wt%, revealing no relationships between TOC content and δ^13^C values. Furthermore, our samples contain thermally immature organic matter and biomarker lipid patterns that are inconsistent with a mainly allochthonous carbon source. More ^13^C-enriched δ^13^C_TOC_ signatures may be the result of alternative mechanisms for carbon assimilation. Carbon-concentration mechanisms or active bicarbonate uptake by prokaryotes can result in smaller values for *ε*_p_ and higher δ^13^C_TOC_ values^67,68^. The emergence of the Ediacaran biota may have significantly expanded marine food webs and stimulated new avenues of microbial heterotrophy, including possible contributions from complex carbon cycling within benthic microbial mats. With additional consideration for the potentially important role of dissolved organic matter^69^ as a carbon source, the breadth of δ^13^C_TOC_ values likely reflects a range of biogeochemical carbon-cycling processes that might be unique to the evolving Ediacaran marine environment and may be related to a phenomenon for which we have no representative modern analogs.

Ancient lipid biomarker assemblages and stable isotope indicators for benthic, microbial mat production in tandem with fixed nitrogen limitation have been found in the early Triassic rocks from South China^70^. In the aftermath of the end-Permian mass extinction at Meishan, there was a large spike in hopane/sterane ratios (up to ca. 60) accompanied by a strong shift in N_org_ isotope signature to values of 0 to –2‰, consistent with bacterial diazotrophy. This is associated with a high signal of 2-methylhopanes (2-methylhopane index up to 33%) and distinctive methylalkanes that point to the proliferation of benthic microbial mats. We do not observe a similar trend in nitrogen isotope signature or biomarker patterns that would obviously point to significant microbial mat input within our Ediacaran data set, although a singular ^15^N-depleted value was found in our samples (Table 1). Therefore, the strong bacterial signal observed in our Baltica samples is likely not predominantly a signature of benthic microbial mats.

### Paleoenvironmental sustenance of Ediacara biota vs. demosponges

**Fig. 3:**
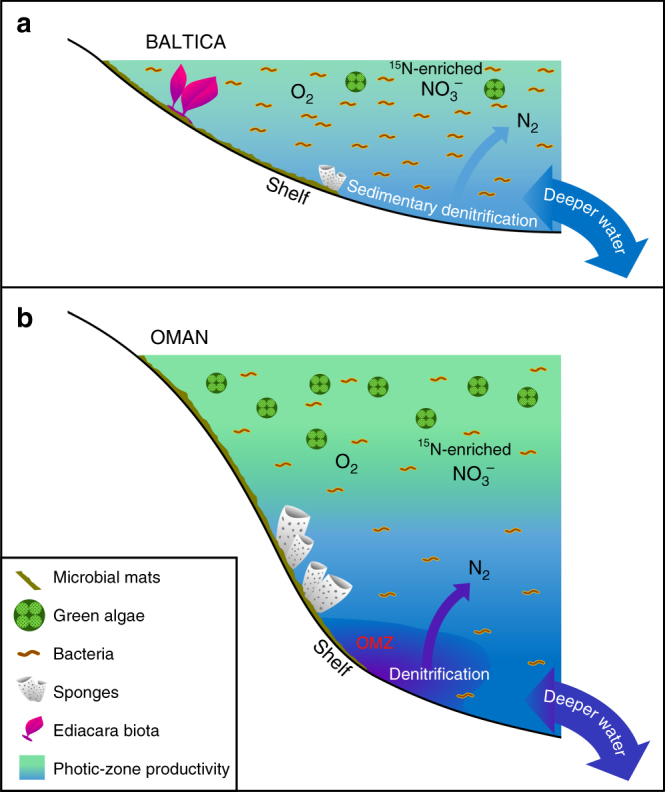
Major differences in low-productivity vs. productive Ediacaran marine environments. Schematic diagrams are shown for **a** extensive oligotrophic and shallow-marine epicontinental basin margins of Baltica often dominated by bacterial productivity where Ediacara soft-bodied fauna flourished and where denitrification and anammox likely were restricted to sediments; and **b** eutrophic and deeper-marine shelf settings of the South Oman Salt Basin, where green algae thrived as a primary producer and demosponges were abundant, but Ediacara soft-bodied fauna was not prominent (with Ediacara biota fossils also absent in correlative Ediacaran outcrops in northern Oman). On productive continental margins, denitrification and anammox likely occurred in both the water column and sediments

The apparent oligotrophic conditions across the epicratonic and continental margin basins of Baltica, as it drifted from high to low latitudes from the late Ediacaran to early Cambrian, might be associated with either limited advection of relatively nutrient-replete deep waters or nutrient depletion, resulting from assimilation and scavenging during transport and deposition across these broad, shallow-marine epicontinental basins (Fig. 3). These basins were episodically isolated from the oceans and developed hypersalinity (e.g., during the Redkino time) and brackish conditions (e.g., during the Kotlin time^71^). Long-term tectonic stability resulted in a low-relief topography of late Ediacaran Baltica, highly susceptible to flooding and inefficient supply of weathering-derived phosphorus. In contrast to Baltica, biomarker studies of the strata from the Huqf Supergroup in Oman revealed an eutrophic ecosystem, rich in microalgae^30,31,59^, but lacking Ediacara biota, even in extensive outcrops in the Oman Mountains and Huqf region, in the inner- to outer-shelf settings^3^. The paleogeography of the South Oman Salt Basin has been reconstructed for the late Neoproterozoic at ~13° from the equator in the southern hemisphere, broadly similar to the Baltica^72^ paleolatitude.

There is no evidence for persistent and extensive anoxia on and around the shallow continental margins of Baltica during the late Ediacaran^11^, and we suggest that the oligotrophic conditions described here were caused by inefficient terrestrial and deep-water nutrient fluxes to these settings, broadly similar to those in oligotrophic environments in modern ocean systems. Indeed, a trace-element geochemical investigation of the Utkina Zavod and adjacent drill cores from the St. Petersburg area suggests that our samples were deposited under oxic conditions^73^. This interpretation is independently supported by our data from the extended hopane (C_31_–C_35_) distributions that tail off sharply in abundance above C_31_ compounds with increasing carbon number, which is characteristic of side-chain degradation of bacteriohopanepolyols during diagenesis under oxic conditions^29^, and also by low-hydrogen indices, as measured by Rock-Eval pyrolysis (all lower than 230 mg/g TOC, and many are below 100 mg/g TOC) for these immature samples due to the formation of recalcitrant kerogen via oxidative degradation and recondensation of lipid-poor primary biomass in locally oxic environments (Table 1). The Ediacaran multicellular organisms that existed in these shallow-marine environments must have had sufficient organic substrates for heterotrophy to meet their feeding needs along with sufficient oxygen and other nutrients to sustain their metabolism. Epicontinental basins of Baltica were likely more persistently oxic than the highly productive settings that fringed oxygen-minimum zones, such as on the middle to the outer shelf of South Oman Salt Basin, where respiration of abundant planktonic biomass would have maintained lower dissolved oxygen below the photic zone.

Ediacara biota and other multicellular organisms living offshore of Baltica would have had to cope with changing food sources (bacterial vs. eukaryotic) as nutrient fluxes varied through time, including small cells and organic detritus in epicontinental basins where bacteria were the dominant primary producers (Fig. 3). Modern coral reef communities survive in tropical oligotrophic settings due to efficient recycling of nutrients, including the generation of a dissolved organic matter (DOM) flux which helps sustain faunal heterotrophy within the reef ecosystem^74^. The establishment of a marine trophic structure with eukaryotic multicellular organisms sustained by feeding on organic detritus had to postdate the global-scale environmental expansion of eukaryotes into diverse marine environments, which occurred through the Tonian–Cryogenian interval (ca. 800–635 Ma), as gauged from biomarker records^15^. We might then expect to discern evidence for significant differences in marine community and trophic structure from locality to locality during the late Ediacaran period, with the local nutrient balance selecting for eukaryote-rich or eukaryote-lean microbial communities and with the progressive expansion of multicellular organisms adding another dimension of complexity at an organismal and community level. Significant regional contrast in phosphate and other nutrient availability in shelf environments is also an expected consequence of a heterogeneous global marine redox structure for the late Ediacaran, prior to the oxygenation of the deep ocean^38^.

The dominance of picoplankton bacterial productivity and associated dissolved organic matter (DOM) degradation products in the Baltica epicontinental basins could have sustained a microbial loop ecosystem in parallel with the conventional trophic structure based on around larger-sized planktonic producers^50^. This may have favored different modes of heterotrophy, including suspension feeding and, possibly, osmotrophy, as a viable feeding strategy for some rangeomorphs, sponges, and other late Ediacaran multicellular organisms^18^ in conjunction with emerging active (motile) heterotrophy^19,20^. Nutrient-limited aquatic systems as a general rule are often dominated by small unicellular phytoplankton and heterotrophic plankton, with bacteria outcompeting eukaryotes, and sustaining low net biomass in oligotrophic marine settings of the modern ocean^49,51^. DOM is an important source of organic nutrients and often controls productivity and net biomass in modern oligotrophic tropical seas, but so is nitrogen and phosphorus co-limitation^75^. Similarly, DOM was also likely an important substrate for sustaining heterotrophic bacteria and a microbial loop in ancient oligotrophic settings. Benthic microbial mats may have been a component of this bacterially dominated food web, and mat grounds have been implicated to enhance the preservation of Ediacara biota in marine settings^7^. While moderate values of 2-methylhopane index were found for a subset of our samples (4–10%, Table 1), very low abundances of methylalkanes relative to *n*-alkanes (Fig. 1), and only trace and sporadic occurrences of carotenoids suggest that microbial mats did not dominate the primary productivity^76^, and that planktonic bacteria and, correspondingly, their DOM breakdown products flourished. The generally low hydrogen-index values (Table 1; especially for Redkino samples), despite the low thermal maturity of the strata and biomarker assemblages, are also consistent with lipid-poor organic input in mainly oxic, shallow-marine marginal settings. Microbial mat occurrences as gauged only from sedimentological textures do not tell us about the balance of eukaryotes (microalgae) to bacteria in any case, nor can they constrain the relative contribution of microbial plankton to overall primary productivity and sustenance of food webs. Microbial mat communities that often contain abundant eukaryotes and the Ara Group carbonates from South Oman with prominent thrombolitic and crinkly laminite facies are a good example of late Neoproterozoic environments with significant microbial mat contribution, yielding abundant sterane signals due to a large contribution of microalgae^30,59^.

The extremely low level of 24-ipc biomarkers suggests that while demosponges were sometimes present, they were sparse in these environments. This could indicate fewer opportunities for smaller filter-feeding animals in competition with the Ediacara biota in these low-productivity settings, insufficient resilience of demosponges against more energetic shallow-marine conditions above the fair-weather wave base, or better adaptability of sponges to low-oxygen conditions dynamically maintained below the photic zone in eutrophic settings (Fig. 3). In total, 24-ipc was not detected in most of the samples of the Redkino Horizon, which also have generally the highest H/St ratios among our sample set, perhaps indicating an ecological change by the Kotlin Horizon depositional time. Alternatively, it is also plausible that sponges did inhabit these settings, but did not produce these diagnostic steroid biomarkers in abundance, although this seems less likely as 24-ipc sterane is among the most commonly detected C_30_ sterane compounds in Ediacaran strata and oils^30,31,33,59,60^ and is also detectable in a subset of our samples (Table 1).

While the persistent oligotrophic marine environments suggested by our data represent localized conditions in the Ediacaran oceans, they were likely not uncommon for Precambrian shallow-marine seaways (Fig. 3). The vast majority of previous Ediacaran biomarker studies have been conducted on organic-rich sedimentary rocks deposited in eutrophic settings and their petroleum products, which generally yield biomarker assemblages consistent with significant microalgal source contribution^44^. Our results highlight the importance of studying a wider variety of depositional environments, including organic-matter-lean strata of appropriate thermal maturity and different lithologies^15,30,34,59^, in order to gain a more accurate picture for the scale of heterogeneity in marine chemistry and ecology from location to location. Despite progressive ocean ventilation and increased chemical weathering and nutrient supply during the breakup of Rodinia and throughout the Ediacaran Period^77,78^, ocean heterogeneity maintained a variety of marine chemical conditions, including nutrient-poor, but habitable environments that fostered metazoan adaptation, competition, and evolution within the global ocean system (Fig. 3). While eutrophic marine shelves hosted demosponges, but often lacked the Ediacara biota, with the South Oman Salt Basin (and the correlative thick Ediacaran outcrops further north in Oman) being a prominent example; counterintuitively, shallow, oxic, and less-productive epicontinental seaways were colonized by the Ediacara biota in preference to demosponges despite DOM and other organic detritus being available locally for feeding. Whether metabolic requirements or environmental selective pressure restricted Ediacaran soft-bodied multicellular biota to these settings remains uncertain; however, our study highlights that Ediacaran oligotrophic settings played a potentially crucial role in the evolution of macroscopic multicellular organisms and marine community ecology.

## Methods

### Sample selection and processing

A total of 29 thermally immature Ediacaran sedimentary rock samples from Russia, Ukraine, and Moldova, encompassing strata from the Redkino, Kotlin, and Lontova horizons, were selected for investigation from the Baltic monocline, Moscow, and Volyn-Podillya Basins. This provided sampling across a wide paleogeographic transect for late Ediacaran marine paleoenvironments of Baltica (Table 1). Details of the geological background, as well as sample locations and lithological information, are provided in Supplementary Notes 1 and 2. All our samples are younger than 560 Ma based on established correlations with the well-dated strata of White Sea and Ural Mountains, Russia and Podillya, Ukraine^25,73^ and thus were deposited during the time interval when shallow-marine waters in the surface mixed layer were redox-stabilized and predominantly oxic^11^, despite evidence for redox instability and anoxic conditions in deeper ocean settings^49^ and in some lower-energy, shallow-marine settings^4^.

Standardized procedures were employed to prevent contamination during the cutting and crushing stages. Rock chips were first trimmed with a clean water-cooled rock saw to remove the outer surfaces. The obtained solid inner portion was sonicated in a sequence of ultrapure water, methanol (MeOH), and dichloromethane (DCM). The cleaned inner-rock fragments were powdered in a zirconia ceramic puck mill using a SPEX 8515 shatterbox, treated between successive samples by powdering two batches of combusted sand (at 850 °C overnight) and rinsing with methanol, DCM, and hexane. As an important control, combusted quartz sand blanks were run parallel with the samples as full analytical procedural blanks. This procedure yielded pristine rock powders for subsequent solvent extraction (see below).

### TOC determination and Rock-Eval pyrolysis

TOC contents were determined at GeoMark Research in Houston, TX. Samples were decarbonated with 5 M HCl for at least 2 h, rinsed through a filtration apparatus to remove the acid, dried at low temperature, and weighed to obtain percent carbonate based on weight loss. They were then combusted on a LECO C230 instrument to measure TOC content. The LECO C230 instrument was calibrated with standards having known carbon contents. Standards were combusted by heating to 1200 °C in the presence of oxygen; both carbon monoxide and carbon dioxide were generated and carbon monoxide was converted to carbon dioxide by a catalyst. The carbon dioxide yield was measured using an IR cell. Combustion of unknowns followed the same procedure and the response per mass unit of unknown was compared to that of the calibration standard. Standards were analyzed every ten samples to check stability and calibrate the analysis. Standard deviation for TOC was 3% from the established value.

Approximately 100 mg of washed, ground (to 60-mesh) whole-rock samples were analyzed with a Rock-Eval II instrument. Measurements include S1: free bitumen content (mg HC/g rock); S2: remaining generation potential (mg HC/g rock); Tmax: temperature at maximum evolution of S2 hydrocarbons (°C); and S3: carbon dioxide yield from organic carbon (mg CO_2_/g rock). The data were generated by heating according to the following parameters: S1: 300 °C for 3 min; S2: 300–550 °C ramping at 25 °C/min, and then held at 550 °C for 1 min; S3: hold at a temperature between 300 and 390 °C. Instrument calibration was achieved using a rock standard with values determined based on a curve calibrated with pure hydrocarbons of varying concentrations. The low values of *T*_max_(ranging from 417 to 443 °C, with mean = 426 °C, *n* = 22) indicate that these rocks are all thermally immature and most of them are at a low thermal maturity stage that is a pre-oil window or (for 16PL outcrop samples only) at an early-to-middle stage within the oil window prior to peak oil generation (Table 1).

### Lipid biomarker analysis

The following methods outlined in Haddad et al.^79^, solvent extractions of rock bitumens were performed on 5–20 g of rock powder per sample using a 9:1 (v/v) DCM:MeOH mixture in a CEM Microwave Accelerated Reaction System (MARS) at 100 °C for 15 min. The total bitumen extract was separated into aliphatic hydrocarbons, aromatics, and polar fractions on a silica gel column, eluting with hexane, a 1:1 (v/v) mixture of DCM and hexane, and a 4:1 (v/v) DCM to MeOH mixture, respectively.

Aliphatic and aromatic hydrocarbon fractions were analyzed to generate total ion chromatograms (Fig. 2) in full-scan mode using a gas chromatography–mass spectrometry (GC–MS) with an Agilent 7890A GC system coupled to an Agilent 5975C inert MSD mass spectrometer. The GC temperature program for full-scan analysis was 60 °C (held for 2 min), heated to 150 °C at 20 °C/min, then to 325 °C at 2 °C/min, and held at 325 °C for 20 min. The GC was equipped with a DB1-MS capillary column (60 m × 0.32 mm, 0.25-µm film thickness) and helium was used as a carrier gas.

To determine accurate molecular biomarker ratios (Table 1), aliphatic hydrocarbons were also analyzed by metastable reaction monitoring (MRM)–GC–MS on a Waters Autospec Premier mass spectrometer equipped with an Agilent 7890A gas chromatograph and DB-1MS coated capillary column (60 m × 0.25 mm, 0.25-µm film) using He as a carrier gas to look at polycyclic biomarker stereoisomer patterns in more detail. The GC temperature was programmed with an initial hold at 60 °C for 2 min, then heating to 150 °C at 10 °C/min rate, followed by heating to 320 °C at 3 °C/min rate, and a final hold for 22 min; analyses were performed via splitless injection in an electron-impact mode, with an ionization energy of 70 eV and an accelerating voltage of 8 kV. MRM ion-pair transitions were used for a suite of biomarker compounds (C_27_–C_35_ hopanes, C_31_–C_36_ methylhopanes, C_19_–C_26_ tricyclic terpanes, C_24_ tetracyclic terpanes, C_21_–C_22_ and C_26_–C_30_ steranes, and C_30_ methylsteranes), which were identified by retention time and published mass spectra and were quantified by comparison of their peak area with that of an added deuterated C_29_ sterane standard [d_4_-ααα-24-ethylcholestane (20R)]. Individual analyte peaks in rock extract hydrocarbon fractions were quantified and found to constitute at least three orders of magnitude larger signal than any peak detected in full-laboratory blank using combusted sand. Procedural blanks with pre-combusted sand typically yielded less than 0.1 ng of individual hopane and sterane compounds per gram of combusted sand.

In addition to the rock bitumen extraction, catalytic hydropyrolysis (HyPy^80^) was used as an important self-consistency check for testing biomarker syngenicity. HyPy was performed on a subset of pre-extracted rock powders (Utkina Zavod 111.6 m, Lugovoe 41 m, and Lugovoe 73 m). This technique involves temperature-programmed heating of samples gradually up to 520 °C in a continuous-flow configuration under high hydrogen gas pressure (15 MPa) to cleave covalent bonds and release the bound molecular constituents, while preserving their structural and stereochemical integrity to a high degree. Because the kerogen is insoluble and immobile, it yields primary biomarker signals immune from contamination due to oil migration or with drilling fluids and represents a key strategy to identify any significant biomarker contaminant in the extractable bitumen phase^80^. Supplementary Figures 1 and 2 show that both the bitumen and kerogen-bound hopanes from the Utkina Zavod (111.6-m) sample exhibit a very immature profile as is the case for the steranes. The bound hopanes yield a slightly less-mature diastereoisomer distribution than the free hopanes due to protection of the bound biomarker pool by covalent binding, which is consistent with the expected bound versus free patterns found for sedimentary rocks of all geological ages^80^. Thus, we are confident that the exceptionally immature polycyclic alkanes found in our bitumen extracts are primary and genuine Ediacaran biomarkers consistent with immature biomarker stereoisomer ratios (Table 1) and with the expected maturity profiles from Rock-Eval pyrolysis parameters (particularly *T*_max_).

### Organic carbon and nitrogen isotope ratios

Nitrogen isotopic analysis of sediments was performed in the Syracuse University GAPP Lab using an automated “nano-EA” that is similar to that described in Polissar et al.^81^. Nano-EA allows for the analysis of small sample sizes that is essential for reliable measurements of N-lean materials or samples for which complete combustion is difficult to achieve. The Syracuse University nano-EA comprises an Elementar Isotope Cube elemental analyzer coupled to an Isoprime Trace Gas analyzer. The Trace Gas analyzer is used for N_2_ trapping and chromatographic focusing prior to the introduction of gas into the Isoprime 100 stable-isotope mass spectrometer. Sample powders were loaded into tin capsules, evacuated, and purged with argon prior to introduction into the EA to remove contamination from atmospheric N_2_. EA conditions were the following: helium purge was set for 45 s, oxidation and reduction reactor temperatures were 1100 °C and 650 °C, respectively, helium carrier gas flow was 150 ml/min, and the O_2_ pulse was set for 60 s. During sample analysis, the full flow of the EA is diverted to automated silica gel-filled cryotrap that is immersed in liquid nitrogen over the duration that N_2_ gas was generated during sample combustion. The N_2_ trap is switched to a low-flow He carrier gas (2 ml/min) via an automated Vici 6-port Valco valve and released to the IRMS through an Agilent CarboBond column (25 m × 0.53 mm × 5 µm). CO_2_ generated during sample combustion was retained in a molecular sieve trap that is integral to the Elementar Isotope Cube EA, which is heated and released to waste after each sample. This eliminates the potential of carryover of CO_2_ and generation of CO in the ion source that would interfere with nitrogen isotope analysis.

Samples were run in triplicate using sequentially larger samples (i.e., 6, 8, and 10 mg) and blank-corrected using Keeling-style plots. International (IAEA N1 ammonium sulfate [0.4 weight‰ N], N2 ammonium sulfate [20.3 weight‰ N], and NIST 1547 peach leaves [2.0 weight‰ N]) and in-house (Messel Oil Shale [7.0 weight‰ N]) reference materials were also run in a similar manner, and in nitrogen quantities that bracketed the N-content of the sample materials. The resulting blank-corrected sample and standard data were calibrated using accepted values for the reference materials applying the scheme described in Coplen et al.^82^. Reproducibility for samples and standards (±0.25‰) approaches that for the reported nitrogen isotope composition of the reference materials (±0.2‰) and is similar to standard EA-IRMS techniques for samples of similar nitrogen content.

Carbon isotope analysis was performed on acidified rock powder residuals using an Elementar Isotope Cube elemental analyzer coupled to an Isoprime 100 stable-isotope mass spectrometer in a conventional format. EA conditions were the following: helium purge was set for 30 s, oxidation and reduction reactor temperatures were 1100 °C and 650 °C, respectively, helium carrier gas flow was 230 ml/min, O_2_ pulse was set for 60 s, and CO_2_ trap was heated to 230 °C to release trapped sample CO_2_. International reference materials (ANU sucrose [−10.4‰] and NIST 1547 peach leaves [−26.0‰]) were used to develop the correction scheme for sample data as described previously^82^. Reproducibility for samples and standards was better than ±0.1‰.

### Data availability

The authors declare that the data supporting the findings of this study are available within the paper and its supplementary information files.

## Electronic supplementary material

Supplementary Information
