# Supplementary material for: Ediacara biota flourished in oligotrophic and bacterially dominated marine environments across Baltica
Source: Nat Commun. 2018 May 4;9:1807. doi: 10.1038/s41467-018-04195-8 (PMC5935690; doi:10.1038/s41467-018-04195-8)
Supplement: Supplementary file 1 — Supplementary Information [file 41467_2018_4195_MOESM1_ESM.pdf]

SUPPLEMENTARY INFORMATION

Ediacara Biota flourished in oligotrophic and bacterially dominated marine environments across Baltica

Pehr *et al*.

Supp1ementary Note 1. Geological background and Ediacaran fossil assemblages of Baltica

Paleomagnetic data track Baltica moving from high to low latitudes in the southern hemisphere between 615 and 550 Ma[S1]. Starting at ~620 Ma, Baltica began rifting from Laurentia and, potentially, Amazonia as part of the breakup of the supercontinent Rodinia. Rifting took place along the modern western edge of Baltica, resulting in the opening of the Iapetus Ocean by 570 Ma. Meanwhile, the eastern margin of Baltica transitioned from a long-lived passive margin to a foreland basin[S2,S3]. Throughout the Ediacaran, deposition across Baltica occurred along the passive margins and in the epicontinental basins, and, at a later stage, in the pro-foreland basin developed along the eastern margin. Study of the Ediacaran strata indicates an extremely low degree of thermal maturation across Baltica, from clay mineralogy[S4] and Rock-Eval pyrolysis (Table 1).

The late Ediacaran strata of Baltica is traditionally divided into two regional biostratigraphic horizons based on extensive study of microfossil records from drill cores across Baltica: Redkino, and Kotlin, whereas the two lowermost horizons of the Early Cambrian (starting with the first appearance of *Treptichnus pedum*) are Rivno and Lontova, in order of oldest to youngest. Extensively developed over Baltica, Redkino is characterized by diverse assemblage of carbonaceous compression macrofossils and soft-bodied biota deposited in marine basins. The younger Kotlin horizon is marked by decreasing diversity and abundance of the soft-bodied biota (although coincidental with the first appearance of *Psammocorallia*) accompanied by emergence of diverse ichnofossils, associated with shallow burrowing organisms, and macroscopic algal assemblages[S5,S6,S7,S8,S9,S10]. This sudden shift in diversity and abundance of soft-bodied biota has been called the “Kotlin Crisis” and has been interpreted as either an extinction event or a migration of soft-bodied biota to distributary channel system caused by the competitive pressure

from bilaterians[S5]. Recently, this two-fold stratigraphic scheme has been challenged with the proposal of an additional short interval of geological time placed between Redkino and Kotlin and tentatively named Belomorian, which would encompass the highest diversity of soft-bodied biota[S11]. In this study we will use the traditional stratigraphy based on the Redkino and Kotlin horizons.

Widespread and abundant occurrences of macroscopic soft-bodied fossils (see Fig. 1) are found in the Ediacaran strata from the Mezen Basin, White Sea region, the South and Central Urals of Russia as well as Podillya of Ukraine, and although these were not described from the Moscow Basin[S12] and Baltic monocline [S13], this might reflect a lack of outcrop with only drill cores available for investigation in the latter areas providing less opportunities to find fossils. Ediacaran strata from the Redkino Horizon contain select Avalon-type fossils, diverse White Sea type fossils, and even some Nama-type biota [S14,S13]. The Kotlin horizon hosts Nama-type fossils[S12,S11]. Noteworthy are abundant occurrences of White Sea-type fossil assemblages from the Redkino Horizon in Podillya outcrops[S15], close to where our own Podillya samples came from. All our samples are younger than 560 Ma based on established correlations with the well-dated strata of White Sea and Ural Mountains, Russia and Podillya, Ukraine[S11,S12] and thus were deposited during the time interval when shallow-marine waters in the surface mixed layer were redox-stabilized and predominantly oxic [S16] despite evidence for redox instability and anoxic conditions in deeper ocean settings[S17] and in some lower-energy, shallow-marine settings [S18].

Supplementary Note 2. Geology of the studied units

Sedimentary rock sample details are given in Supplementary Table 1. In the St. Petersburg area of Russia, the Redkino Horizon is represented by the locally developed Staraya Russa

Formation, which averages 50 m in thickness. It consists of mudstones and siltstones. The Staraya Russa Formation records a full transgressive-regressive cycle, potentially formed in a submarine, delta-fan setting and low-energy environment of sea shoals. The overlying Vasileostrovskaya Formation of the Kotlin Horizon has a basal unconformity in the Utkina Zavod drill core, but not in the Lugovoe drill core[S19]. Its lower part consists of silty mudstones, while its upper part is composed of laminated mudstones with fine, horizontal, and wavy bedding. These sediments are interpreted to have been deposited under generally oxic conditions along the coast with weak currents and in stagnant depressions in the sublittoral environments[S19].

In the Gavrilov Yam-1 drill core of the Moscow Basin of Russia, siltstones of the Reshminskaya Formation that belong to the Kotlin Horizon and the lower part of the Early Cambrian Lezhskaya Formation that belongs to the Lontova Horizon were sampled[S20]. The sediments were accumulated in epicontinental seaways with depths straddling the storm wavebase under predominantly oxic conditions.

In the Volyn area of the northwestern Ukraine (Volyn-Polissye Basin), the drill cores 4504 and 4529 containing mainly siltstones of the Kanyliv Group (undivided in this area, but correlated to the Upper Kotlin Horizon) were sampled. Mudstones of the Roznychi Formation of the Mohyliv-Podilsky Group that belong to the Upper Redkino Horizon were sampled in the drill core 4592.

The presently separate Volyn-Polissya Basin and Podillya Basin, developed in the western part of Ukraine and extending across its border to Moldova, belonged to the same Late Ediacaran Volyn-Podillya epicontinental basin extending to the continental margin to the south. They are now separated by the older Precambrian crystalline basement, uplifted during the orogenic event along the southern margin of Baltica in the earliest Paleozoic. In the Podillya Basin, the drill core

3628 from the northernmost part of the Podillya Basin, Ukraine and outcrops in Moldova near the border with Ukraine were sampled. The samples from the drill core 3628 are from the Lomozov Beds of the Mohyliv Formation, the Lyadova Beds of the Yaryshiv Formation, and the Kalyus Beds of the Nagoryany Formation, which belong to the lower, middle, and upper intervals of the Redkino Horizon, respectively. The outcrop samples from Moldova are from the upper Redkino Kalyus Beds of the Nagoryany Formation. The 20-m-thick Lomozov Beds contain thin, interbedded dark-grey mudstones and fine-grained sandstones; in middle part of the beds thick intervals of arkosic gravelites occur. The Lomozov Beds constitute the oldest strata hosting Ediacara Biota macrofossils within the succession; they have the highest macrofossil diversity through the Ediacaran succession of the Volyn-Podillya Basin[S21]. The 25-m-thick Lyadova Beds contain greenish-grey and brown thin-bedded micaceous mudstones with a gradual lower contact. The beds contain films of algal (or, possibly, fungal) origin and an assemblage of microphytofossils, typical for the Mohyliv Formation[S22]. The 50-m-thick Kalyus Beds are represented by homogeneous, dark-grey thin-bedded mudstones. The beds contain in the middle part up to 15 levels of phosphorite concretions and have gradual lower contact, marked by lenses and interbeds of fine-grained calcareous sandstone. The beds include two 4- and 5.5-m-thick levels, at the top and bottom, rich in Vendotaenian algae.

Supplementary Table 1. Sample details and locations for Ediacaran rocks used in this study

Drillcore # and its location	Sample depth	Name of Formation/ Group	Regional stratigraphic horizon	Lithology
Northwestern part of the Volyn area (close to city Kovel'), Ukraine				
4504; village Tur, next to lake Tur; 51.680686 N, 24.282324 E	~200 m	Kanyliv Gr.	Upper Kotlin	Mica-rich, chocolate-brown siltstones
4529; village Poliske; 51.477429N, 24.514081E	~195 m	Kanyliv Gr.	Upper Kotlin	Grey siltstones interlayered with sandstones and mudstones
4529; village Poliske; 51.477429N, 24.514081E	~207 m	Kanyliv Gr.	Upper Kotlin	Grey siltstone interlayered with sandstone and mudstone
4592; village Kamianukha; 51.237161 N, 25.718928 E	~166 m	Roznychi Fm.; Mohyliv-Podilsky Gr.	Upper Redkino	Dark-brown mudstones with mica-rich siltstones

Moscow Basin, Russia				
Gavrilov Yam-1; 57.31111111 N, 39.91361111 E	1860-1870 m	Lezhskaya Fm.	Lontova	Grey siltstone
Gavrilov Yam-1: 57.311111 N, 39.913611 E	2018.0 – 2018.3 m	Reshminskaya Fm.	Kotlin	Grey siltstone
Saint-Petersburg area, Baltic monocline, Russia				
Utkina Zavod; 59.852778 N, 30.507222 E	64.15 m	Vasileostrovskaya Fm.	Kotlin	Green, silty mudstone
Utkina Zavod; 59.852778 N, 30.507222 E	71.67 m	Vasileostrovskaya Fm.	Kotlin	Green/grey, silty mudstone with siderite
Utkina Zavod; 59.852778 N, 30.507222 E	87.0 m	Vasileostrovskaya Fm.	Kotlin	Green, silty mudstone
Utkina Zavod; 59.852778 N, 30.507222 E	111.6 m	Vasileostrovskaya Fm.	Kotlin	Green, silty mudstone

Utkina Zavod; 59.852778 N, 30.507222 E	119.9 m	Vasileostrovskaya Fm.	Kotlin	Green to dark-grey, silty mudstone
Utkina Zavod; 59.852778 N, 30.507222 E	124.6 m	Vasileostrovskaya Fm.	Kotlin	Green, silty mudstone
Utkina Zavod; 59.852778 N, 30.507222 E	127.8 m	Vasileostrovskaya Fm.	Kotlin	Green, silty mudstone
Utkina Zavod; 59.852778 N, 30.507222 E	152.8 m	Vasileostrovskaya Fm.	Kotlin	Green, silty mudstone
Utkina Zavod; 59.852778 N, 30.507222 E	153.5 m	Vasileostrovskaya Fm.	Kotlin	Green to grey, silty mudstone
Utkina Zavod; 59.852778 N, 30.507222 E	162.1 m	Vasileostrovskaya Fm.	Kotlin	Green, silty mudstone
Utkina Zavod; 59.852778 N, 30.507222 E	171.4 m	Vasileostrovskaya Fm.	Kotlin	Green, silty mudstone

Lugovoe #13; 60.695000 N, 30.195833 E	41.0 m	Vasileostrovskaya Fm.	Kotlin	Green, silty mudstone
Lugovoe #13; 60.695000 N, 30.195833 E	44.0 m	Vasileostrovskaya Fm.	Kotlin	Green, silty mudstone
Lugovoe #13; 60.695000 N, 30.195833 E	47.0 m	Vasileostrovskaya Fm.	Kotlin	Green, silty mudstone
Lugovoe #13; 60.695000 N, 30.195833 E	71.0 m	Staraya Russa Fm.	Redkino	Green mudstone/siltstone
Lugovoe #13; 60.695000 N, 30.195833 E	73.0 m	Staraya Russa Fm.	Redkino	Green, silty mudstone
Lugovoe #13; 60.695000 N, 30.195833 E	75.0 m	Staraya Russa Fm.	Redkino	Green, silty mudstone

Northern part of the Podillya Basin, between cities of Khmelnitsky and Rivne (Ukraine)				
3628, village Denysivka, river Semenivka 49.921217N, 26.458906E,	226.5 m	Mohyliv-Podilsky	Uppermost Redkino	Light-brown mudstones with phosphorites
3628, village Denysivka, river Semenivka 49.921217N, 26.458906E	291 m	Mohyliv-Podilsky	Middle Redkino	Light-grey micaceous mudstone with interlayers of siltstone and sandstone
3628, village Denysivka, river Semenivka 49.921217N, 26.458906E	332 m	Mohyliv-Podilsky	Middle to low Redkino	Greenish-grey and grey mudstone with thin interlayers of siltstone
Southern part of the Podillya Basin, right bank of the Dniester river (Moldova)				
16PL-11, village Naslavcea 48.465779N, 27.583773E	Surface outcrop	Mohyliv-Podilsky	Upper Redkino	Dark-grey phosphorite-bearing mudstone

16PL-18, village Naslavcea, open pit near the Dniester river 48.480442N, 27.562998E	Surface outcrop	Mohyliv-Podilsky	Upper Redkino	Dark-grey phosphorite-bearing mudstone
16PL-22, village Naslavcea, open pit near the Dniester river 48.480970N, 27.563809E	Surface outcrop	Mohyliv-Podilsky	Upper Redkino	Dark-grey phosphorite-bearing mudstone

Supplementary References

S1. Klein, R., Salminen, J. & Mertanen, S. Baltica during the Ediacaran and Cambrian: A paleomagnetic study of Hailuoto sediments in Finland. *Precambrian Research* **267,** 94–105 (2015).

S2. Sliaupa, S., Fokin, P., Lazauskiene, J. & Stephenson, R. A. The Vendian-Early Palaeozoic sedimentary basins of the East European Craton. *Geological Society, London, Memoirs* **32,** 449–462 (2006).

S3. Pease, V. *et al.* Baltica in the Cryogenian, 850–630Ma. *Precambrian Research* **160,** 46–65 (2008).

S4. Aksenov, E.M., & Volkova, S.A. Volcanogenic-Sedimentary Horizons of Redkino Suite of Valday Series. *Doklady Akademii Nauk SSSR* **188**, 635-639 (1969).

S5. Kolesnikov, A. V., Marusin, V. V., Nagovitsin, K. E., Maslov, A. V. & Grazhdankin, D. V. Ediacaran biota in the aftermath of the Kotlinian Crisis: Asha Group of the South Urals. *Precambrian Research* **263,** 59–78 (2015).

S6. Burzin, M. B. Late Vendian (Neoproterozoic III) Microbial and Algal Communities of the Russian Platform: Models of facies-dependent distribution, evolution and reflection of basin development. *Rivista Italiana di Paleontologia e Stratigrafia (Research In Paleontology and Stratigraphy)* **102,** (1996).

S7. Martin, M. W. *et al.* Age of Neoproterozoic Bilatarian Body and Trace Fossils, White Sea, Russia: Implications for Metazoan Evolution. *Science* **288,** 841–845 (2000).

S8. Veis, A. F., Vorob'eva, N. G. & Golubkova, E. Y. The early Vendian microfossils first found in the Russian plate: Taxonomic composition and biostratigraphic significance. *Stratigr. Geol. Correl.* **14,** 368–385 (2006).

S9. Vorob'eva, N. G., Sergeev, V. N. & Knoll, A. H. Neoproterozoic Microfossils from the Northeastern Margin of the East European Platform. *Journal of Paleontology* **83,** 161–196 (2009).

S10. Marusin, V. V., Grazhdankin, D. V. & Maslov, A. V. Redkino stage in evolution of Vendian macrophytes. *Dokl. Earth Sc.* **436,** 197–202 (2011).

S11. Grazhdankin, D. V. & Maslov, A. V. The room for the Vendian in the International Chronostratigraphic Chart. *RGG* **56,** 549–559 (2015).

S12. Grazhdankin, D. Patterns of Evolution of the Ediacaran Soft-Bodied Biota. *Journal of Paleontology* **88,** 269–283 (2014).

S13. Podkovyrov, V. N., Maslov, A. V., Kuznetsov, A. B. & Ershova, V. B. Lithostratigraphy and geochemistry of Upper Vendian–Lower Cambrian deposits in the northeastern Baltic monocline. *Stratigr. Geol. Correl.* **25,** 1–20 (2017).

S14. Laflamme, M., Darroch, S. A. F., Tweedt, S. M., Peterson, K. J. & Erwin, D. H. The end of the Ediacara biota: Extinction, biotic replacement, or Cheshire Cat? *Gondwana Research* **23***,* 558–573 (2013).

S15. Ivantsov et al. Upper Vendian macrofossils of Eastern Europe. Russian Academy of Sciences, Moscow, 1–146 (2015)

S16. Johnston, D. T. *et al.* Late Ediacaran redox stability and metazoan evolution. *Earth and Planetary Science Letters* **335-336,** 25–35 (2012).

S17. Sperling, E. A. *et al.* Statistical analysis of iron geochemical data suggests limited late Proterozoic oxygenation. *Nature* **523,** 451–454 (2015).

S18. Marusin, V. V., Grazhdankin, D. V. & Maslov, A. V. Redkino stage in evolution of Vendian macrophytes. *Dokl. Earth Sc.* **436,** 197–202 (2011).

S19. Podkovyrov, V.N., Maslov, A.V., Kuznetsov, A.B. & Ershova, V.B. 2017, Lithostratigraphy and geochemistry of Upper Vendian–Lower Cambrian deposits in the northeastern Baltic monocline. *Stratigraphy and Geological Correlation* **25**, 1–20 (2017).

S20. Vinogradov, V. I. *et al*. Rb–Sr and K–Ar Characteristics of Upper Vendian Clayey Rocks in the Russian Platform: *Lithology and Mineral Resources* **40**, 332–352 (2005).

S21. Ivantsov *et al.* Upper Vendian macrofossils of Eastern Europe. Russian Academy of Sciences, Moscow, 1–146 (2015)

S22. Velikanov, V.A., Aseeva, E.A., and Fedonkin, M.A., The Vendian of Ukraine. Excursion Guide. Kiev: Naukova Dumka, 1983, 1–162 [in Russian].

SUPPLEMENTARY FIGURES

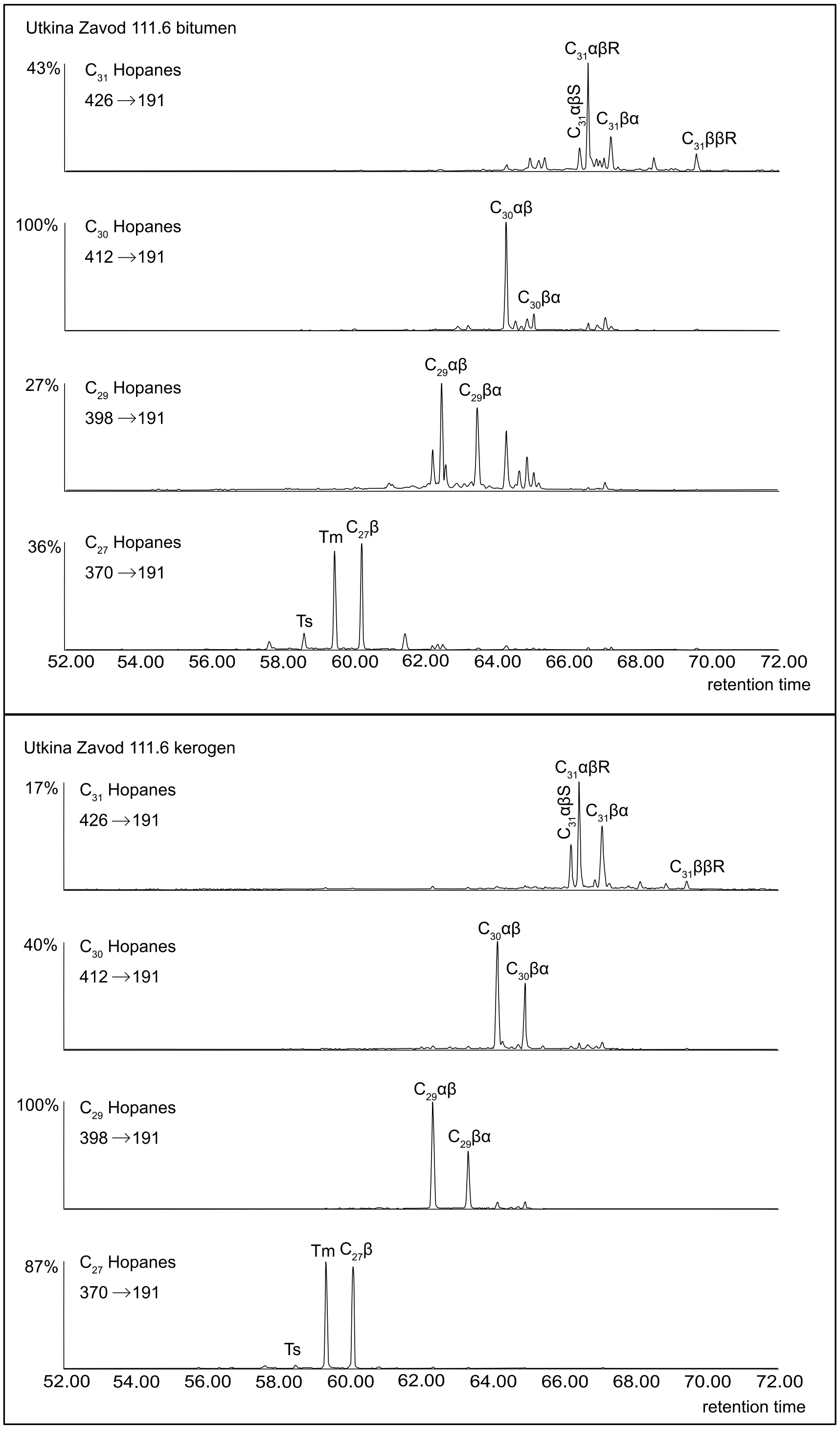

Supplementary Figure 1. MRM-GC-MS chromatograms for the free and kerogen-bound aliphatic hydrocarbon fractions generated from sample Utkina Zavod (111.6m depth) with C_{27} – C_{31} hopanes labeled. *Top* traces show *free* hopane distributions from bitumen extraction; *bottom* chromatograms show *bound* hopanes generated from the insoluble organic matter (kerogen) using catalytic hydropyrolysis (HyPy).

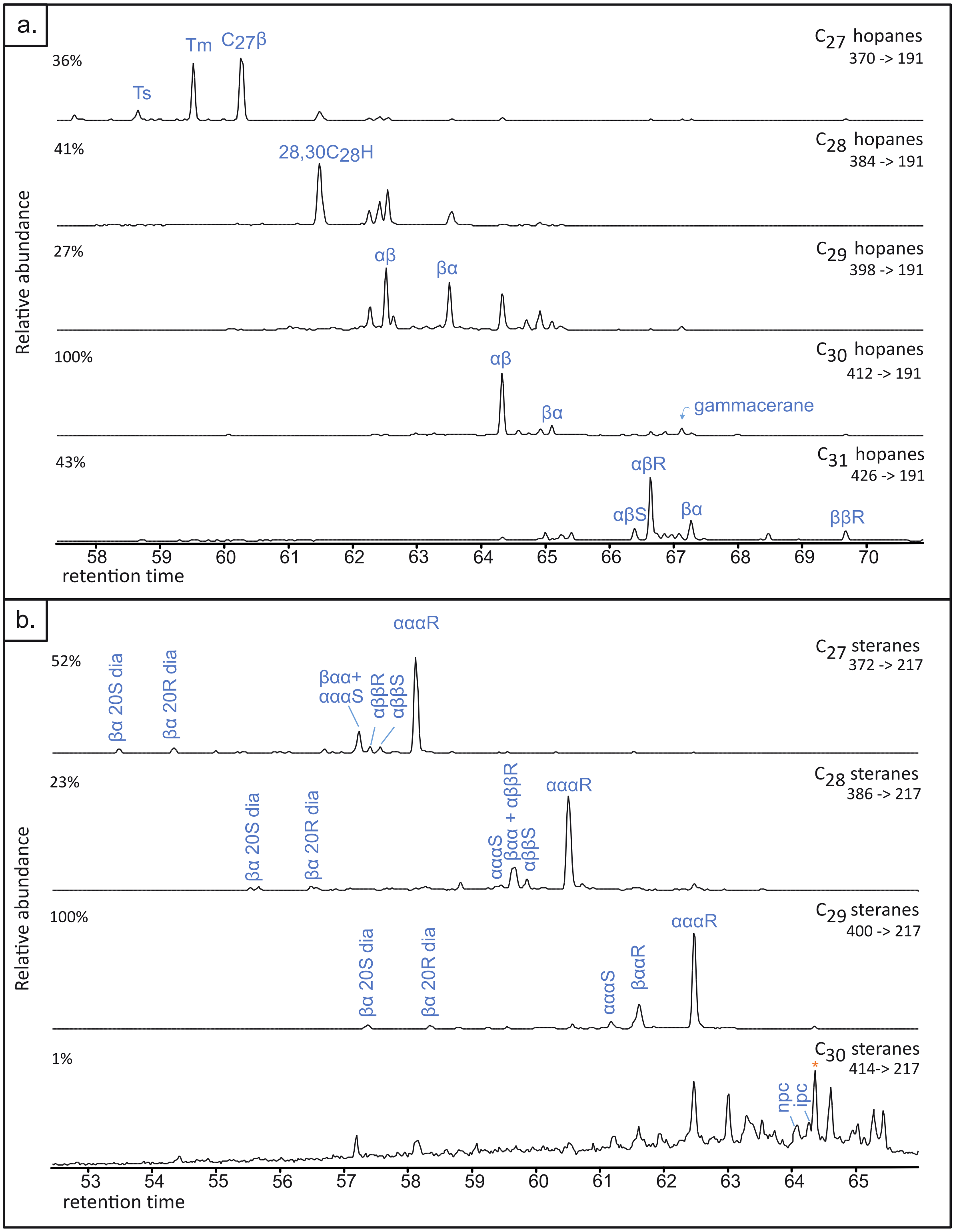

Supplementary Figure 2. MRM-GC-MS chromatograms for the extractable aliphatic hydrocarbon fraction for sample Utkina Zavod (111.6m depth). *Top* chromatograms show C_{27} – C_{31} hopanes labeled; *bottom* chromatograms show C_{27} – C_{30} steranes labeled.
